# MEPD: medaka expression pattern database, genes and more

**DOI:** 10.1093/nar/gkv1029

**Published:** 2015-10-07

**Authors:** Juan I. Alonso-Barba, Raza-Ur Rahman, Joachim Wittbrodt, Juan L. Mateo

**Affiliations:** 1 Department of Computing Systems, University of Castilla-La Mancha, Albacete, 02071, Spain; 2 Centre for Organismal Studies, University of Heidelberg, Heidelberg, 69120, Germany

## Abstract

The Medaka Expression Pattern Database (MEPD; http://mepd.cos.uni-heidelberg.de/) is designed as a repository of medaka expression data for the scientific community. In this update we present two main improvements. First, we have changed the previous clone-centric view for *in situ* data to a gene-centric view. This is possible because now we have linked all the data present in MEPD to the medaka gene annotation in ENSEMBL. In addition, we have also connected the medaka genes in MEPD to their corresponding orthologous gene in zebrafish, again using the ENSEMBL database. Based on this, we provide a link to the Zebrafish Model Organism Database (ZFIN) to allow researches to compare expression data between these two fish model organisms. As a second major improvement, we have modified the design of the database to enable it to host regulatory elements, promoters or enhancers, expression patterns in addition to gene expression. The combination of gene expression, by traditional *in situ*, and regulatory element expression, typically by fluorescence reporter gene, within the same platform assures consistency in terms of annotation. In our opinion, this will allow researchers to uncover new insights between the expression domain of genes and their regulatory landscape.

## INTRODUCTION

Medaka (*Oryzias latipes*) is already an established model organism in developmental biology (1,2). Key properties for this status are extra-embryonic development and the transparency of the embryo. In addition, the possibility of having hundreds of embryos per day makes medaka amenable to high-throughput screens of gene expression. The Medaka Expression Pattern Database (MEPD) was initiated already more than 10 years ago (3,4) with the aim of serving as a central repository for gene expression patterns to the scientific community. At that time the medaka genome sequence was not yet available and therefore all the information was based on expressed sequence tags (EST).

In the meantime, the medaka genome was sequenced (5) using a shotgun approach to the Hd-rR inbred line with 10.6-fold coverage. The N50 value is 5.1Mb excluding gaps. This assembly is labelled as *draft* version, but at the time of writing this manuscript there is already a preliminary version of a new assembly that aims to be ‘near-complete’ (Kiyoshi Naruse, personal communication).

The availability of the genome sequence together with the gene annotation accomplished by the ENSEMBL team (6) implies a major change in the structure of MEPD. We have now implemented this change by shifting the previous clone-centric view to a gene-centric view.

However, this is not the only update. Medaka is as well a good model organism to study transcriptional regulation and the expression domain of regulatory elements, namely promoters and enhancers. Already there are many works published using medaka to analyse the spatio-temporal activity domain of this kind of elements (7–10). Other model organisms serve similar purpose and there are as well online databases making available these data like REDfly (11) for the fruit fly, Expression disruption screen (12) or Enhancer screen (http://www.upo.es/CABD/EnhancerScreen/) for zebrafish and the Vista enhancer browser (13) for mouse. Nonetheless there is not yet any site that integrates both sets of expression data, genes and regulatory elements, using the same vocabulary and ontology for annotation. This is the motivation that led us to incorporate this second part of expression information into MEPD. With this update we envision that, as the data hosted here grow, it will represent a valuable resource to analyse the logic and rules of transcriptional gene regulation.

## DATABASE CONTENT

**Figure 1.**
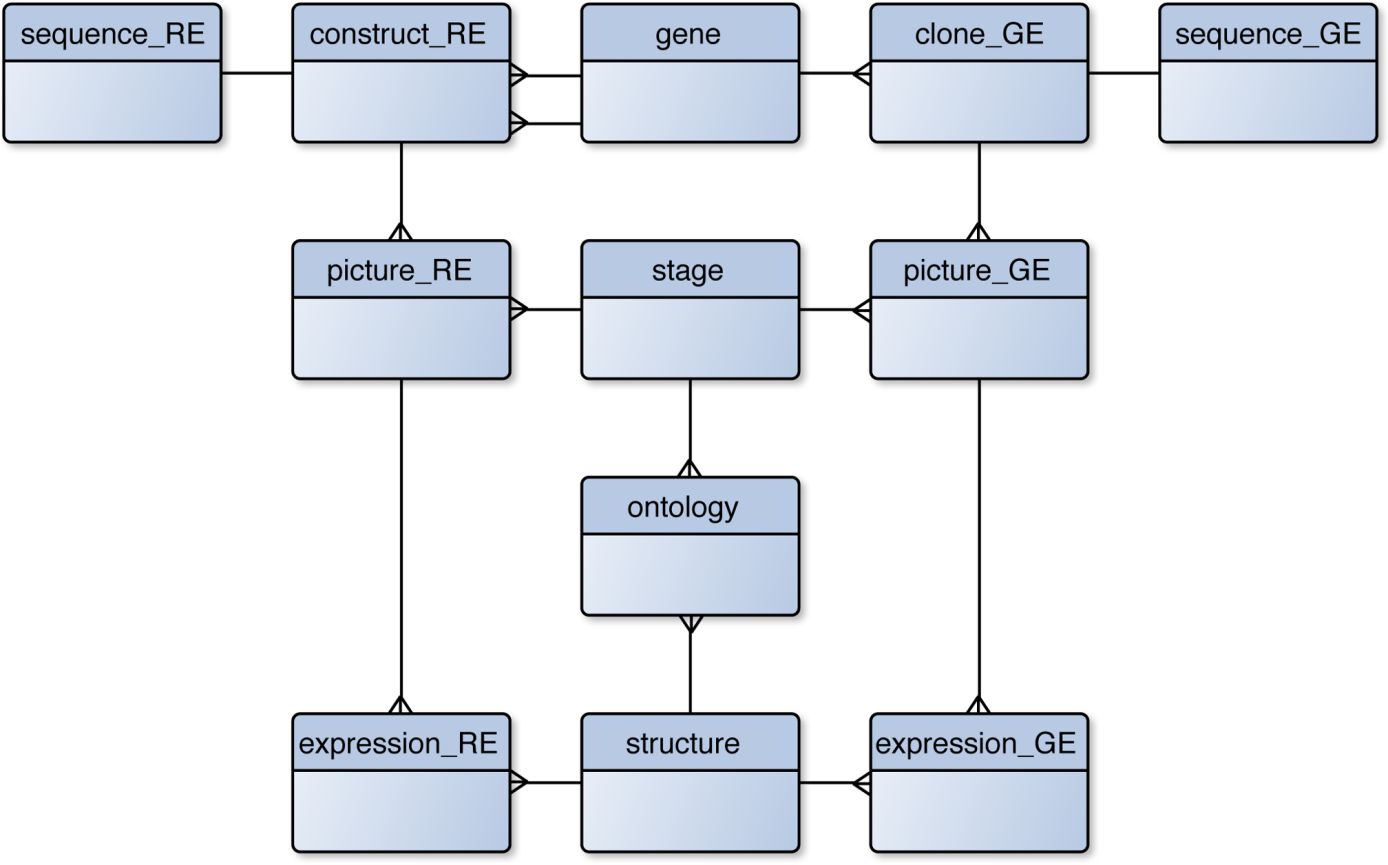
Simplified entity–relationship model of MEPD. The structure of the database is maintained from previous releases (see tables with ‘GE’ suffix for *in situ* data), but we have added the tables for regulatory element data, with ‘RE’ suffix as a clone of those for gene expression data. For simplicity the tables are shown without fields.

We have maintained the basic structure of the database from previous versions. There is, however, one main change related to the tables to accommodate regulatory expression data. We have replicated the structure containing the information of gene expression pattern with new tables for regulatory elements. For *in situ* data, tables with GE suffix in Figure 1, the information is distributed over the tables clone, sequence, picture and expression. The same structure fits now the information for regulatory elements, suffix RE in this case, substituting clone for construct. As represented in Figure 1, a construct is linked to two genes, which represent the closest up- and down-stream genes from the locus of each element. This assignment is done with respect to the direction in which the regulatory sequence is tested. If this element was not tested in a specific orientation, then we consider the forward strand independently on which sequence, forward or reverse strand, is added to the database. In case of a sequence overlapping a gene or an intragenic element this gene will be set both as up- and down-stream.

This association to genes is only for searching purpose, i.e. being able to retrieve expression patterns of regulatory elements near to a given gene. However, it is not straightforward to validate the interaction of a regulatory element and its target gene(s), therefore this should not be taken, in general, as the genes over which the enhancer/promoter acts.

With the availability of the genome sequence and gene annotation, it is not anymore necessary to relay on blast hits to identify the target gene of an *in situ* probe, as it was in previous versions. Therefore, we have eliminated the blast and cluster tables. Thus, now the gene is the centre entity of the database. We use ENSEMBL (6) as our reference for gene annotation, although for clone sequences not matching a gene annotated in this database we make use of an automatic annotation of RNA-seq data at different stages of development (Mateo et al. in preparation).

Taking advantage of the orthology relationship defined by the ENSEMBL database between medaka and zebrafish, we have included a new table containing this information. With that, from the result page of gene or regulatory element expression, it is possible to access a link to the corresponding gene in the Zebrafish Model Organism Database (ZFIN) (14) for comparison. This functionality can be very useful to easily identify conserved or diverging expression patterns of orthologous genes between these two fish species.

At the moment of writing this manuscript, MEPD contains expression data for 947 genes and 7476 images, from 623 and 3863, respectively, in the previous update. For regulatory elements the current content is 56 elements with 69 pictures. Already now it is possible to illustrate the power of using MEPD, for instance comparing the precise overlapping expression pattern of the *RX2* gene (15) and its direct upstream regulatory element (promoter) (16).

## DATA ACCESS

The MEPD data are stored in a MySQL database. The access to these data is done through a Java web application using JavaServer Faces and JBoss RichFaces technology, which is running on a Tomcat server. The access to the data for gene or regulatory element expression is done via separate forms, although in both cases the same filters can be applied, namely: gene, annotated anatomical structure and stages.

The data submission can be done also through the web interface. This functionality is only available to registered users, but anyone can register an account. We have improved significantly the data entry forms with a tabulated view of the clones or constructs. This view allows sorting and multi-column filtering by gene name or ID and the name of the clone or construct. After selecting one of the items in the table, the information related to that item is shown in the right panel. Using this panel it is possible to modify or add information in the corresponding fields.

In order to ease the submission of large amount of expression data we have also implemented a bulk upload function. In this case, the user can fill the required information in a spread sheet. This sheet can be sent to us together with the corresponding pictures. We will perform a manual check of the data for consistency and coherence and then include them on behalf of the submitter.

In the ‘Links’ section of MEPD online (http://mepd.cos.uni-heidelberg.de/mepd/forms/links.jsf) it is possible to download the MEPD user manual and a template spread sheet to upload bulk data.

## AUTOMATIC ANNOTATION

We have used an automated pipeline to associate each clone sequence to the proper ENSEMBL gene. This pipeline is based on blat alignments (17) of the sequences in MEPD to the cDNA sequences of the genes annotated in ENSEMBL, or to our unpublished annotation based on RNA-seq data. In the future we will use the same pipeline to update the gene references when new versions of the medaka genome assembly or the ENSEMBL gene annotation are published.

For entry of new data, the user is responsible to assign the proper gene name and ID to each record. However, on the process of an automatic update, conflicting cases, in which the genes assigned by the user and the automatically assigned are not the same, we will perform a manual evaluation. In this evaluation we will contact, if possible, the responsible user.

## FUTURE DIRECTIONS

With the new improved data entry interface and bulk updates we expect that the volume of data uploaded to MEPD will increase significantly. Specially, we aim at hosting an amount of expression data for regulatory sequences comparable to that of gene expression. This information will be very important for researchers willing to create fish transgenic lines with specific spatio-temporal expression domains.

In order to ease this task, we are planning to include also in MEPD information about stable transgenic lines from laboratories willing to share them with the rest of the community.

As mentioned before, we foresee that combining gene and regulatory element expression patterns MEPD, and medaka as model organism, can become a primary resource for deciphering and understanding transcriptional regulation in vertebrates.
